# Screening of Acetylcholinesterase Inhibitors by Capillary Electrophoresis with Oriented-Immobilized Enzyme Microreactors Based on Gold Nanoparticles

**DOI:** 10.3390/molecules29010118

**Published:** 2023-12-24

**Authors:** Jian Zhang, Yuanyuan Li, Lin Chen, Zhihong Zheng, Chunye Liu

**Affiliations:** 1 School of Pharmacy, Xi’an Medical University, Xi’an 710021, China; zhangjian@xiyi.edu.cn (J.Z.); lyy555569@163.com (Y.L.); tjngzcl@foxmail.com (L.C.); zzh935338689@163.com (Z.Z.); 2 Institute of Medicine, Xi’an Medical University, Xi’an 710021, China

**Keywords:** capillary electrophoresis, oriented-immobilized enzyme microreactor, acetylcholinesterase, gold nanoparticles, enzyme inhibitor screening

## Abstract

A facial and efficient method for the screening of acetylcholinesterase (AChE) inhibitors by capillary electrophoresis was developed. Based on the specific affinity of concanavalin A (Con A) for binding to the glycosyl group of AChE, enzyme molecules were oriented-immobilized on the surface of gold nanoparticles (AuNPs@Con A@AChE). Then, these modified nanoparticles were bounded to the capillary inlet (about 1.0 cm) by electrostatic self-assembly to obtain the oriented-immobilized enzyme microreactor (OIMER). Compared to an IMER with a free enzyme, the peak area of the product obtained by the OIMER increased by 52.6%. The Michaelis–Menten constant (*K_m_*) was as low as (0.061 ± 0.003) mmol/L. The method exhibits good repeatability with a relative standard deviation (RSD) of 1.3% for 100 consecutive runs. The system was successfully applied to detect the IC_50_ values of donepezil and four components from Chinese medicinal plants. This work demonstrates the potential of this method as a low cost, simple, and accurate screening method for other enzyme inhibitors.

## 1. Introduction

Alzheimer’s disease (AD) is the most common form of dementia, which will affect over 130 million people worldwide by 2050 [1]. Many studies suggest that AD is associated with a low level of acetylcholine (ACh). In AD patients, levels of ACh can decrease by up to 90 percent [2]. Acetylcholinesterase (AChE) is the enzyme responsible for ACh hydrolysis. Inhibiting this enzyme could prevent the normal breakdown of ACh. While an ACh deficit is just one of the many factors (for example, the deposition of the amyloid-β (Aβ) peptide in the brain) contributing to AD, the current market-approved drugs for alleviating AD symptoms are AChE inhibitors, including donepezil, tacrine, and galanthamine [3,4]. Therefore, the development of efficient methods for screening AChE inhibitors would greatly aid in diagnosis and in finding a cure for AD.

Capillary electrophoresis (CE) is an analytical technique that is widely used for efficient microfluidic separation and is now a powerful platform for online analysis of enzymatic reactions [5,6]. In CE, the immobilized enzyme microreactor (IMER) is an efficient miniaturized enzyme analytical system. In an IMER, the enzyme is immobilized onto the inner surface at the capillary inlet for the enzymatic reaction, while the remaining part of the capillary is used for the separation of analytes. IMERs present essential advantages, including low sample consumption, reduced enzyme costs, enhanced enzyme stability, and the possibility to reuse enzymes without any complicated purification procedure [7]. In practice, the capillary exhibits a small effective surface area and may not be able to provide a conversion rate high enough for the enzyme reaction.

Thus, efforts have been made to find a strategy to overcome this shortcoming. For instance, enzyme-modified nanoparticles or porous-layer-modified columns have been applied to improve the effective surface area due to the vast surface-to-mass ratio of nanoparticles and the unique microporous structure of porous-layer-modified columns [7,8,9,10]. A layer-by-layer assembly bioreactor is another choice to enhance the stability and loading amount of enzymes due to the multilayers of enzyme immobilization [11,12]. However, these methods usually yield randomly bound enzymes, making it challenging to control the orientation and distribution of the enzymes. Furthermore, the random orientation and distribution may lead to reduced accessibility of the active moieties, thereby resulting in a loss of enzyme activity compared to its free form, whereas the oriented immobilization of enzymes could overcome the above shortcomings. Moreover, oriented-attachment enhances the effective surface area, thus increasing the enzyme loading amount [13,14,15].

In this work, the advantages of the vast surface-to-mass ratio of nanoparticles and oriented immobilization were combined to further improve the loading amount and the activity of enzymes. First, concanavalin A (Con A) was bonded to gold nanoparticles (AuNPs@Con A) [16]. It is well documented that thiol groups (–SH) present in protein molecules strongly bind to AuNPs, contributing to the high stability of protein-AuNPs conjugates [17]. Then, AChE molecules were modified with AuNPs@Con A to obtain an oriented-immobilized enzyme (AuNPs@Con A@AChE) based on the specific (bio)affinity of Con A to the glycosyl group of AChE [13]. Finally, nanoparticles of AuNPs@Con A@AChE assembled on the positively charged inner wall of the inlet end due to electrostatic interactions, resulting in the oriented-immobilized enzyme microreactor (OIMER).

## 2. Results

### 2.1. Characterization of Nanoparticles

**Figure 1:**
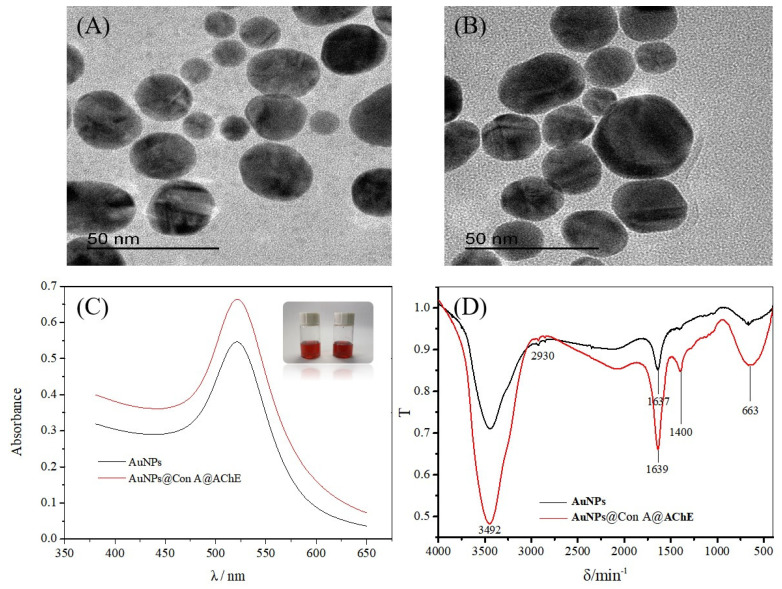
TEM images of (**A**) AuNPs and (**B**) AuNPs@Con A@AChE, (**C**) the UV-visible absorption spectrum of AuNPs (black line) and AuNPs@Con A@AChE (red line), and (**D**) the FT-IR spectra of AuNPs (black line) and AuNPs@Con A@AChE (red line). Insets of (**C**) are the photos of AuNPs (left) and AuNPs@Con A@AChE (right), respectively.

Transmission electron microscopy (TEM) images illustrate the regular sphere shape and good dispersibility of the AuNPs (Figure 1A) and the AuNPs@Con A@AChE (Figure 1B). The size and zeta potential of the AuNPs and AuNPs@Con A@AChE nanoparticles were analyzed on a Malvern Zetasizer Nano zen3600 instrument. The results showed that the particle size of the AuNPs and the AuNPs@Con A@AChE was (34.39 ± 0.26) nm and (102.8 ± 0.42) nm, respectively. The Zeta potential of the AuNPs and AuNPs@Con A@AChE was measured as (−44.5 ± 1.25) mV and (−69.5 ± 1.12) mV, respectively.

The UV-visible absorption spectrum was also used to confirm the existence of AChE on the AuNPs surface (Figure 1C). The maximum absorption of AuNPs@Con A@AChE is 522 nm, which has a redshift from the 520 nm of AuNPs.

Fourier transform infrared (FT-IR) spectra of the AuNPs and AuNPs@Con A@AChE are shown in Figure 1D. AuNPs@Con A@AChE has a very strong absorption band at 1400 cm^−1^, which is characteristic of C–N absorption in an amide group. In contrast, there was no absorption at the opposite wave number in the FT-IR spectrum of the AuNPs. 

The magnitude of electroosmotic flow (EOF) in capillary columns depends on the net surface charge density of chargeable groups. So, the EOF value is usually used for characterizing the modification of the inner surface of a capillary. In this work, different capillaries were characterized by testing the EOF, including bare, 1.0 cm long hexadimethrine bromide (HDB)-coated and 1.0 cm long AuNPs@Con A@AChE-coated capillaries. The EOF value decreased from 0.0177 cm^2^·V^−1^·min^−1^ to 0.0150 cm^2^·V^−1^·min^−1^ after the attachment of HDB onto the capillary surface, indicating the attachment of positively charged HDB on the inner surface. On the other hand, the EOF value increased to 0.0153 cm^2^·V^−1^·min^−1^ following the adsorption of 1.0 cm long AuNPs@Con A@AChE coating.

### 2.2. Enzyme Loading Amount of the OIMER

**Figure 2:**
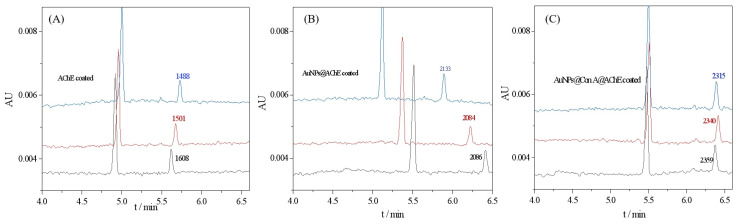
Electropherograms of online analysis of enzymatic reactions with (**A**) free AChE-coated, (**B**) AuNPs@AChE-coated, and (**C**) AuNPs@Con A@AChE-coated capillaries. Conditions: coated column, with dimensions of 75 μm i.d (375 μm o.d) × 60.2 cm (50.0 cm to detection window); injection, 0.5 psi × 10 s; running voltage, −20 kV; running buffer, PBS (20 mmol/L, pH 8.0); capillary temperature, 25 °C; and detection wavelength, 214 nm. The concentrations of ATCh were all 0.05 mmol/L. The electropherograms marked with three colors (black, blue, and red) represent three repeatable assays.

Enzyme loading in the bioreactor mediated by AuNPs is expected to be much greater than that prepared from a free enzyme solution because enzyme molecules can be enriched on AuNPs with a vast surface-to-mass ratio [8]. The oriented immobilization of enzymes could enhance the effective surface area, thus further increasing the enzyme loading amount. Furthermore, the orientation distribution may lead to increased accessibility of the active moieties, thereby resulting in an improvement in enzyme activity. Therefore, the bioreactor was first evaluated in terms of the AChE activity of the bioreactor. To evaluate the enzyme loading capacity and activity, the peak area of the product thiocholine (TCh) obtained with free enzyme-coated, AuNPs@AChE-coated, and AuNPs@Con A@AChE-coated capillaries were measured under the same CE conditions. The concentration of the substrate acetylthiocholine iodide (ATCh) was fixed at 0.05 mmol/L. The electropherograms of the assay are presented in Figure 2. The results show that the peak area of TCh obtained with the AuNPs@Con A@AChE and AuNPs@AChE-coated capillaries increased by 52.6% (*n* = 3) and 37.1% (*n* = 3) compared to the free AChE-coated capillary, respectively. This fact indicated that an oriented immobilization procedure of enzymes, coupled with AuNPs, caused significant improvement in enzyme loading amount and activity.

### 2.3. Activity of the OIMER

**Figure 3:**
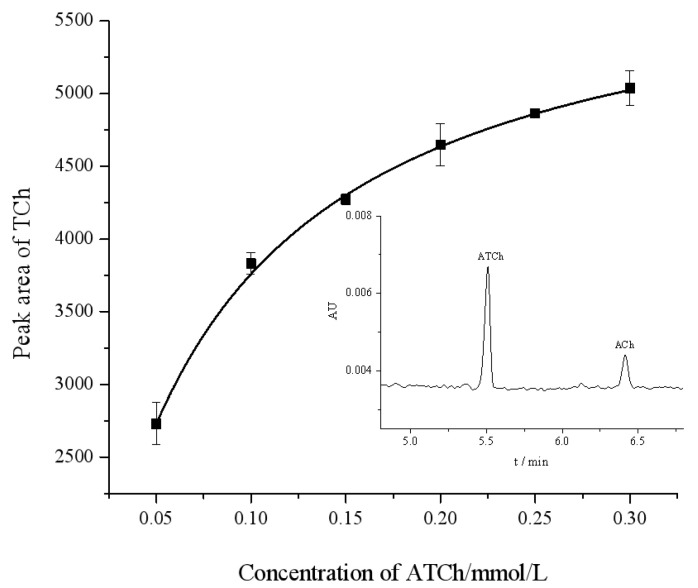
Michaelis–Menten diagram for assay of OIMER of AChE using CE. The standard error of each data point is derived from three repeatable measurements. The inset figure is a typical electropherogram. Electrophoresis conditions are the same as described in Figure 2.

The Michaelis–Menten constant (*K_m_*) is an important parameter that indicates the affinity of the enzyme for a substrate, and its value is inversely proportional to affinity. The *K_m_* was determined by the Michaelis–Menten Equation (1). We measured the peak area of the product TCh at different concentrations of the substrate ATCh in the range of 0.05–0.30 mmol/L. By applying a nonlinear regression in the Michaelis–Menten diagram (Figure 3), *K_m_* and *V_max_* for the OIMER were determined to be *K_m_* = (0.061 ± 0.003) mmol/L and *V_max_* = (6040.566 ± 58.129) mmol/L/min, respectively.

### 2.4. Repeatability and Stability of the OIMER

**Figure 4:**
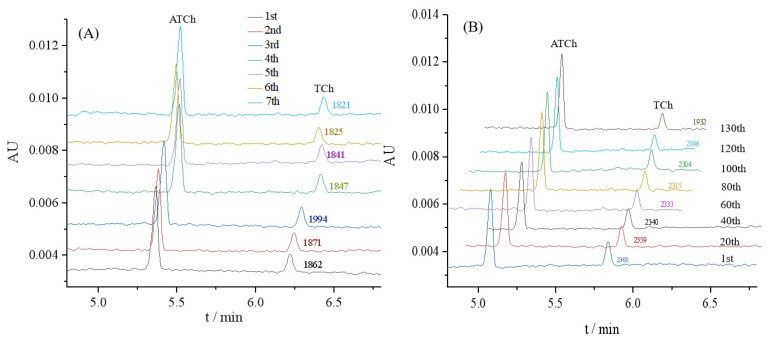
Electropherograms for (**A**) repeatability and (**B**) stability evaluations of OIMER for online analysis of enzymatic reaction. Conditions of electrophoresis are the same as described in Figure 3. In repeatability and stability evaluations, 0.05 mmol/L substrate ATCh (with 1.5 μg/mL gastrodin) and 0.05 mmol/L substrate ATCh (without any inhibitor) were used as samples, respectively.

The repeatability was investigated to show the performance of the proposed OIMER. The relative standard deviation (RSD) values of the ACh peak area were measured to evaluate the repeatability. Seven assays were performed using one OIMER. In these studies, 0.05 mmol/L substrate ATCh (with 1.5 μg/mL gastrodin) was injected into the CE capillary with the OIMER at the inlet end (Figure 4A). The results show that the RSD of the ACh peak area (*n* = 7) was 3.2%, thereby indicating the good reproducibility of the CE separation.

Meanwhile, the stability of the OIMER was assessed. The AuNPs@Con A@AChE-coated capillary column was continuously employed for the online analysis of the enzymatic reaction to evaluate the stability of the OIMER. As shown in Figure 4B, the peak areas tended to decrease as the number of assays increased over the 100th. It suggests that the OIMER can be used for approximately 100 consecutive runs (about 1500 min, with a RSD of 1.3%). The relative activity of the enzyme remained over 81% after 130 times consecutive assays.

### 2.5. Enzyme and Inhibition Assays Using the OIMER

**Figure 5:**
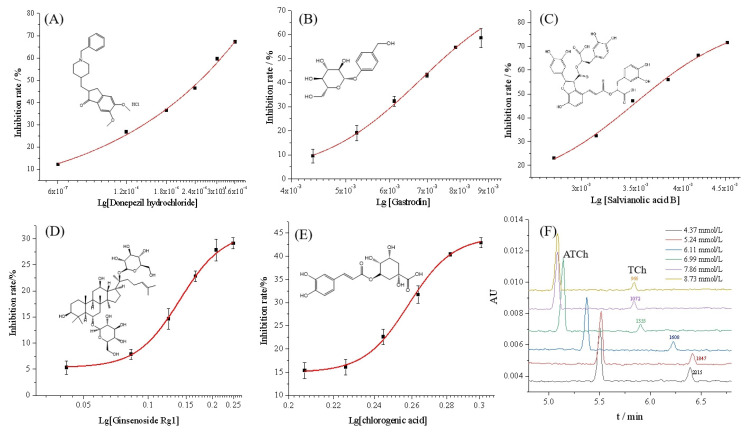
Inhibition curves of (**A**) donepezil hydrochloride, (**B**) gastrodin, (**C**) salvianolic acid B, (**D**) ginsenoside Rg1, (**E**) chlorogenic acid, and (**F**) the typical electropherograms of online analysis of enzymatic reaction in the presence of inhibitor (gastrodin) at different concentrations. Conditions of electrophoresis are the same as those described in Figure 2.

By using the proposed OIMER, the inhibition rate of donepezil hydrochloride was measured in the range of 0.6–3.6 μmol/L, while keeping the substrate concentration constant (0.05 mmol/L). The inhibition plot of donepezil hydrochloride is shown in Figure 5A and the half-maximal inhibition concentration (IC_50_) value was calculated to be (0.81 ± 0.12) μmol/L, which is consistent with the reference UV value (0.82 ± 0.13) μmol/L. The inhibition constant, *K_i_,* could be calculated using the Cheng–Prusoff equation (Equation (4)). According to the *K_m_* of the OIMER (0.061 ± 0.003 mmol/L), the calculated *K_i_* was (0.44 ± 0.01) μmol/L. The results show that the proposed method achieved satisfactory accuracy and reliability.

Four components from Chinese medicinal plants, including gastrodin, salvianolic acid B, ginsenoside Rg1, and chlorogenic acid, were prepared at a series of concentrations and determined under the same conditions. As illustrated in Figure 5B–E, the inhibition rate data (calculated with Equation (2)) fitted well to the Logistic equation, and the inhibition plots were established accordingly for each compound. Figure 5F shows the typical electropherograms for the online analysis of enzymatic reaction in the presence of the inhibitor (gastrodin) at different concentrations. According to the corresponding calibration curves, the IC_50_ values of gastrodin, salvianolic acid B, ginsenoside Rg1, and chlorogenic acid were calculated to be (6.79 ± 0.57), (3.49 ± 0.10), (0.141 ± 0.003), and (0.258 ± 0.002) mmol/L, respectively. Additionally, the reference UV method and Equation (3) were used to calculate the inhibition rate. The IC_50_ of the four components were found to be (6.37 ± 0.18), (3.47 ± 0.11), (0.149 ± 0.007), and (0.255 ± 0.002) mmol/L, respectively. The results indicate no significant difference between the two methods. For the OIMER-CE system, the calculated *K_i_* values of gastrodin, salvianolic acid B, ginsenoside Rg1, and chlorogenic acid were (3.74 ± 0.03), (1.92 ± 0.01), (0.08 ± 0.0002), and (0.14 ± 0.0001) mmol/L, respectively. According to the results obtained, the order of inhibitory potency was ginsenoside Rg1 > chlorogenic acid > salvianolic acid B > gastrodin. This finding also showed the potential of the proposed method for screening inhibitors and for evaluating the activity of different enzymes.

## 3. Discussion

In this study, TEM, size and zeta potential, UV-visible absorption spectrum, and FT-IR were used to characterize the nanoparticles. As shown in Figure 1A,B, the different TEM images and the increased particle size indicate the successful immobilization of AChE and Con A onto the AuNPs microparticles. The negatively charged AuNPs@Con A@AChE can attach to the positively charged inner surface of the capillary by electrostatic self-assembly. The color and the maximum absorption of AuNPs@Con A@AChE have no obvious changes after 30 days, which indicate the stability of AuNPs@Con A@AChE (Figure 1C). Furthermore, the characteristic C–N absorption of the amide group in the FT-IR spectrum of AuNPs@Con A@AChE also showed that AChE had been successfully modified onto the surface of the AuNPs (Figure 1D). To further demonstrate the successful immobilization of enzymes on the capillary, the EOF was measured. Variations in the EOF value in different capillaries can be attributed to the charge of HDB or AuNPs@Con A@AChE.

Kinetic studies were performed with the proposed OIMER method, and the Michaelis–Menten plot (Figure 3) was constructed. The Michaelis–Menten plot shows that the color intensity increased as the concentration of the ATCh increased from 0.05 to 0.30 mmol/L. Compared with the IMERs that, based on advanced materials in the literature, have a *K_m_* value of (1.24 ± 0.05) mmol/L and *V_max_* = (2.18 ± 0.09) mmol/L/min [7] (with porous polymer coating), 1.12 mmol/L and 2.87 mmol/L/min (with chitosan and glutaraldehyde-modified magnetic nanoparticles MnFe_2_O_4_, [18]), or 0.77 ± 0.06 mmol/L and 16.29 mmol/L/min (with densely packed UiO-66-NH_2_ nanocrystal coating, [19]), the OIMERs demonstrated a notably lower *K_m_* and a bigger *V_max_* value (0.061 ± 0.003 mmol/L and 6040.566 ± 58.129 mmol/L/min). This finding suggests a much higher affinity between the enzyme and the substrate of the OIMER. The greater affinity may result from increased accessibility of the substrate to the enzyme’s active site, facilitating the diffusion of the substrate towards the enzyme’s active sites and thus accelerating the affinity and binding rate. 

More importantly, the OIMER avoids the need for advanced materials and a complex preparation process. For instance, the fabrication of a UiO-66-NH_2_ nanocrystal coating will take about 10 h [19]. To obtain the chitosan and glutaraldehyde-modified magnetic nanoparticles MnFe_2_O_4_, at least three main synthesis steps are required (taking approximately 17 h), and extra neodymium magnets are needed to assemble the nanoparticles on the inner surface of capillary [18]. However, the preparation process of the OIMER is simple and time-saving due to electrostatic self-assembly. It takes about 1 h to fabricate the AuNPs@Con A@AChE nanoparticles and 10 min to attach them on the inner surface of the capillary.

The developed OIMER-CE enzyme analysis system showed good repeatability, stability, accuracy, and strong practicability for screening the inhibitors of an enzyme. It fulfilled various requirements, including reusability, ease of use, low cost, rapid analysis, high repeatability, and high stability. The decrease in the enzymatic activity and changes in the migration time after using for 130 consecutive assays may be due to the leaching of enzymes. Fortunately, it is very easy to renew the OIMER once its efficiency becomes poor because the coating process is reversible.

## 4. Materials and Methods

### 4.1. Materials

The fused silica capillary, with dimensions of 75 μm i.d. (375 μm o.d.) × 60.2 cm (50.0 cm to detection window), was purchased from Yongnian Reafine Chromatogram Equipment Co., Ltd. (Yongnian, China) and used for IMER fabrication and CE separation. AChE (220 U/mg), acetylthiocholine iodide (ATCh, ≥98.0%), and donepezil hydrochloride (≥98.0%) were all purchased from Macklin Biochemical Technology Ltd. Co. (Shanghai, China). Bovine serum albumin (BSA, ≥98.0%) was purchased from Aladdin Reagents (Shanghai) Co., Ltd. (Shanghai, China). Hexadimethrine bromide (HDB, ≥94.0%) was obtained from Sigma-Aladdin Chemical Co. (Shanghai, China). Concanavalin A (Con A) was from Baisha Biotechnology Co. (Anhui, China). Salvianolic acid B and Gastrodin were obtained from Shanghai Yuanye Biotechnology Co. (Shanghai, China). All the reagents were of analytical grade, and deionized water was used throughout all the experiments. All solutions were prepared in deionized (DI) water. All the solutions were stored in the refrigerator at 4 °C and filtered with 0.45 μm pore membrane filters prior to use.

### 4.2. Synthesis of AuNPs@Con A@AChE Nanoparticles

**Figure 6:**
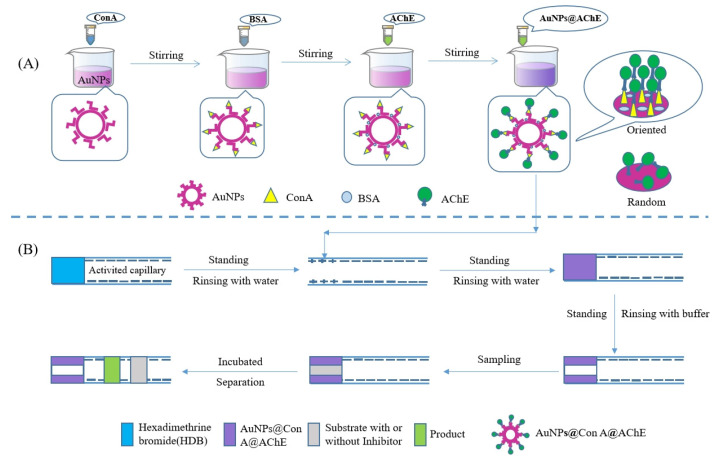
Schematic illustration of (**A**) the preparation of AuNPs@Con A@AChE nanoparticles and (**B**) OIMER and the enzyme/inhibitor activity assay.

The gold nanoparticles (AuNPs) solution was synthesized in a similar fashion to that reported in our studies [20] with minor adjustments. Briefly, 3.5 mL HAuCl_4_ (25.4 mmol/L) was added into 96.5 mL deionized water and heated until the boiling point. Subsequently, 10 mL of 38.8 mmol/L trisodium citrate was added into the boiling solution, and the resulting solution was continuously boiled for 10 min after a red mixture was obtained. The stirring continued for 15 min at room temperature to obtain the AuNPs solution. Then, 1.0 mg Con A was added to the 5 mL AuNPs solution. After stirring for 20 min at room temperature, 1.0 mg BSA was added and stirred for 20 min continually. Finally, 20 μL AChE solution (11 U/mL) was added. The mixture was stirred for 30 min constantly to obtain the modified nanoparticles (AuNPs@Con A@AChE). Here, BSA was used to block nonspecific binding sites on the surface of nanoparticles (Figure 6A).

### 4.3. Fabrication of the OIMER

Figure 6B shows the fabrication procedure of the OIMER. Prior to the first use, the capillary was sequentially rinsed with 1 mol/L NaOH, deionized water, 1 mol/L HCl, and deionized water for 30, 10, 15, and 10 min, respectively. After the capillary was dried with N_2_ gas, it was then used to immobilize the enzymes using the following procedure. First, the cationic polyelectrolyte hexadimethrine bromide solution (HDB, 1 mg/mL) was pushed into the capillary using pressure (0.5 psi × 10 s) and incubated for 5 min. The remaining HDB was removed by rinsing with deionized water. In this way, a positively charged coating with a length of about 1.0 cm was created. Subsequently, a plug of AuNPs@Con A@AChE solution was pushed into the capillary (0.5 psi × 10 s). After standing for 5 min, the negatively charged nanoparticles could be electrostatically adsorbed on the positively charged section of the capillary. Finally, the unbound nanoparticles were flushed out from the column with PBS buffer (20 mmol/L, pH 8.0) and the OIMER was created.

Once the function of an OIMER becomes poor, it can be regenerated as follows. The capillary is rinsed with NaCl (1 mol/L), HCl (0.1 mol/L), and NaOH (0.1 mol/L) for 10, 15, and 20 min, respectively. Then, the aforementioned coating procedure can be repeated.

For comparison, the random IMERs were also prepared by the same electrostatic assembly procedure with AuNPs@AChE solution or free AChE solution.

### 4.4. Online Assay Using the OIMER

All CE experiments were carried out using a Beckman P/ACE MDQ capillary electrophoresis system (Beckman Kurt, Pasadena, CA, USA) equipped with a UV detector working under 25 °C with a liquid temperature control system. Before practice, the capillary was balanced with PBS buffer solution (20 mmol/L, pH 8.0) for 5 min. The substrate solution (0.05 mmol/L ATCh, with or without inhibitor) was injected into the capillary using pressure at 0.5 psi for 10 s and incubated for 1 min. Finally, a −20 kV voltage was applied to separate the product and the unreacted substrate (Figure 6B). The analytes, including the substrate and product, were detected at 214 nm. The activity of the OIMER or the inhibitors was directly determined by measuring the peak area of the product (TCh). The inhibition rate was calculated using the Equation (2).

### 4.5. Reference UV Method

The UV spectrophotometric assay was selected as the reference method and performed according to the methodology described by Ellman et al. [21] with some adaptations. The reaction mixture, which contained 100 μL ATC (0.05 mmol/L, with or without inhibitors), 50 μL AChE (11 U/mL), and 2.50 mL PBS buffer (10 mmol/L, pH 7.4), was incubated for 10 min at 37 °C using a water bath. Then, 1.0 mL SDS (0.04 g/mL) was added. Finally, the absorbance at 208 nm was measured using a UV-vis spectrometer. For the UV method, the ACE-inhibition rate was calculated according to Equation (3).

### 4.6. Related Formulas and Definitions

In order to evaluate the activity of the OIMER, the Michaelis–Menten constant *K_m_* was determined using the Michaelis–Menten Equation (1):

(1) $$V=\frac{V_{max}[S]}{[S]+K_{m}}$$

where *V* and *V*_max_ represent the initial rate and the maximum rate of the enzymatic reaction, respectively, and [*S*] is the substrate concentration. The initial rate *V*, which can be represented by the peak area of the product in this study, was determined to vary ATCh concentrations while other concentrations remained fixed.

(2) $$\mathrm{Inhibition}\;\mathrm{rate}=\frac{S_{0}-S}{S}\;\times \;100\%\;$$

where *S* and *S*_0_ are the peak areas obtained with substrates containing different concentrations of inhibitor or without inhibitor, respectively. The capillary was flushed with PBS buffer (20 mmol/L, pH 8.0) for 5 min between successive injections.

(3) $$\mathrm{Inhibition}\;\mathrm{rate}=\frac{A_{0}-A_{s}}{A_{0}-A_{blank}}\;\times \;100\%\;$$

where *A*_0_, *A_s_*, and *A_blank_* represent the absorbance of a control system without inhibitor being present, the system with a given concentration of inhibitor, and the PBS buffer (10 mmol/L, pH 7.4), respectively.

Inhibition constant *K_i_* can be calculated using the Cheng–Prusoff equation [22]:

(4) $$K_{i}=\frac{{IC}_{50}}{1+\left(\left[S\right]/K_{m}\right)}\;\times \;100\%\;$$

where *K_m_* and [*S*] represent the Michaelis–Menten constant and the concentration of substrate, respectively.

## 5. Conclusions

The AChE could be easily oriented-immobilized on the Con A-modified AuNPs. The AuNPs@Con A@AChEs were assembled on the inner wall at the inlet end of a CE capillary modified by HDB, producing an in-line OIMER. The OIMER showed a high enzymatic activity and stability. It improves the affinity between the enzyme and substrate, resulting in a lower *K_m_* value without the need for advanced materials. The value of the *K_m_* was (0.061 ± 0.003) mmol/L, which was much lower than that reported in the literature. The results suggested that the significant enhancement of the enzyme efficiency was achieved by oriented enzyme immobilization. The use of a highly effective OIMER, in combination with the proposed CE quantification of product ACh, allowed for accurate and reliable AChE inhibitor screening. Compared with the traditional Ellman method, the OIMER-CE method is time and solvent-saving. Moreover, the most interesting feature of the method is the reusability of the enzyme. The OIMER can be used for approximately 100 consecutive assays. The proposed screening method was applied for the evaluation of donepezil hydrochloride and four components from Chinese medicinal plants. It was proved to be accurate and easy to carry out. Furthermore, such a method can be extrapolated and applied to other enzyme activity analyses or inhibitor screenings.

## Data Availability

The datasets are publicly available at NCBI with Sequence Read Archive (SRA) accession.
